# A new species of *Aegyptobia* and redescription of *Tenuipalpusszarvasensis* Bozai, 1970 (Acari, Tenuipalpidae)

**DOI:** 10.3897/zookeys.785.27684

**Published:** 2018-09-19

**Authors:** Jenő Kontschán, Géza Ripka

**Affiliations:** 1 Plant Protection Institute, Centre for Agricultural Research, Hungarian Academy of Sciences, H-1525 Budapest, P.O. Box 102, Hungary Centre for Agricultural Research, Hungarian Academy of Sciences Budapest Hungary; 2 National Food Chain Safety Office, Directorate of Plant Protection, Soil Conservation and Agri-environment, Department of Pest Management Development and Coordination, H-1118 Budapest, Budaörsi út 141–145, Hungary National Food Chain Safety Office Budapest Hungary

**Keywords:** tenuipalpids, mites, flat mite, taxonomy, Hungary

## Abstract

A new tenuipalpid mite species, *Aegyptobiabozaii***sp. n.**, is described from Central-Hungary on leaves of the endemic Hungarian statice Limoniumgmeliniisubsp.hungaricum (Plumbaginaceae) based on females, nymphs and larva. The previously described endemic flat mite, *Tenuipalpusszarvasensis* Bozai, 1970 is redescribed. This species had been treated as a junior synonym of *Tenuipalpuscheladzeae* Gomelauri, 1960, but our new investigation shows that the two species are not the same.

## Introduction

Tenuipalpid mites are a diverse group of plant-feeding mites found in most regions of the world. Several species are pests, especially within *Brevipalpus*, but pest species are also found in *Dolichotetranychus*, *Raoiella* and *Tenuipalpus*. The family has received considerable attention in some parts of the world, but the majority of the Central European countries have been scarcely investigated. Hungary is no exception, with only 19 recorded species (Kontschán and Ripka 2017). However, numerous rare and endemic flat mites might remain undiscovered in natural ecosystems in Hungary; and disturbed ecosystems may harbor several unrecorded or possibly new species.

The aim of our paper is to describe a new tenuipalpid species from Hungary and to redescribe the endemic Hungarian flat mite *Tenuipalpusszarvasensis* Bozai, 1970, which we also remove from its synonymy with *Tenuipalpuscheladzeae* Gomelauri, 1960 by Mitrofanov and Strunkova (1979).

## Material and methods

Specimens of the new species (*Aegyptobiabozaii* sp. n.) were collected in a pasture close the border of the village Farmos (Central-Hungary) from the leaves of an endemic Hungarian plant (Limoniumgmeliniisubsp.hungaricum). The specimens were placed into lactic acid for a week and then slide-mounted in Keifer’s F-medium (in 2014) and Hoyer medium (in 2017). The holotype and some paratypes of the new species are stored in the Hungarian Natural History Museum and other paratypes in the Arachnida collection of the Natural History Museum of Geneva (Switzerland).

The type specimens of *Tenuipalpusszarvasensis* Bozai, 1970 were loaned from the Hungarian Natural History Museum.

All specimens were investigated using a Leica 1000 scientific microscope; the illustrations were made with the aid of a drawing tube on this microscope. Pictures were made with a VHX-5000 with 20–200× objective (Keyence Co., Osaka, Japan) digital microscope. All measurements and scales are given in micrometers.

## Result

### Aegyptobia bozaii sp. n.

Taxon classificationAnimaliaProstigmataTenuipalpidae

http://zoobank.org/CF433BD9-1455-4EE2-84F3-DFF3ABCC858C

Figures 1 , 2–8 , 9 , 10–15 , 16 , 17–21 , 22 , 23–26

#### Material examined.

Holotype: female, Hungary, Pest county, Farmos, 47°22'30"N, 19°52'08"E, 10 m a.s.l, from the leaves of the Hungarian statice, Limoniumgmeliniisubsp.hungaricum, 2 August 2014, Ripka, G. coll. Paratypes: one female, three deutonymphs, three protonymphs and one larva, locality and date same as for holotype. Other paratypes: four females, Hungary, Farmos, 47°22'30"N, 19°52'08"E, 10 m a.s.l, from the leaves of Limoniumgmeliniisubsp.hungaricum, 2 August 2017, Kontschán, J. and Ripka, G. coll.

#### Diagnosis

(based on female). Tarsal claws uncinate. Anterior margin of prodorsal shield with paired projections, prodorsum weakly sculptured, with few irregular lines. Opisthosoma with polygonal reticulation. Propodosomal and opisthosomal setae smooth and simple; seta *f2* present. Length of dorsal setae 6–14. Dorsal opisthosomal pores close to *e1*. Rostrum extending to middle of tibia I. Genital flap smooth. Intercoxal area between *3a* and *4a* smooth.

#### Description

(females; n = 6). Idiosoma reddish-brown (Figure 27), oval in shape, body measured from *v2* to *h1* 240–245; from tip of rostrum 260–266; width 143–147 near setae *sc2*; distance between setae *sc2* 120–125; length of legs I–IV (without coxa), leg I 105–110, leg II 80–87, leg III 72–78, leg IV 80–88.

**Figure 1.**
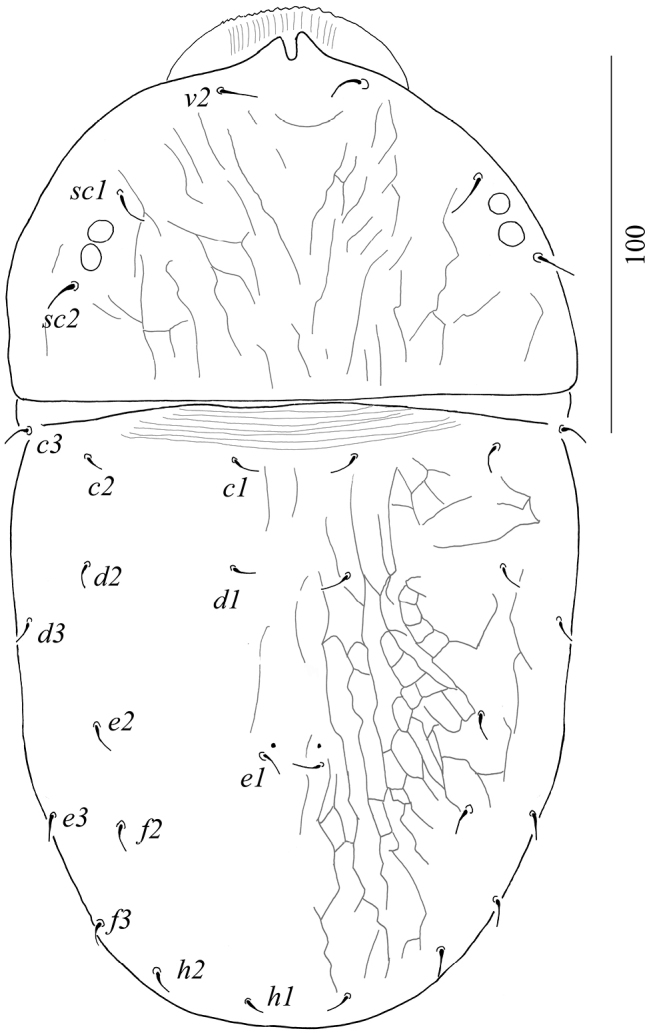
Dorsal view of *Aegyptobiabozaii* sp. n., holotype, female.

Dorsum (Figure 1): Anterior margin of prodorsal shield with paired projections, depth of notch 7–8. Propodosoma finely lineate. Opisthosoma with polygonal reticulations; polygons longitudinally elongate medially, transversally elongate anterolaterally. Propodosomal and opisthosomal setae simple and smooth. Opisthosomal pores present close to *e1*. Prodorsal setae *v2* shorter than half distance between their bases. Length of dorsal setae: *v2* 11–12, *sc1* 12–14, *sc2* 11–13, *c1* 8–9, *c2* 8–9, *c3* 9–10, *d1* 9–10, *d2* 8–9, *d3* 7–8, *e1* 8–9, *e2* 7–8, *e3* 8–9, *f2* 7–8, *f3* 6–8, *h1* 6–7, *h2* 7–8.

**Figures 2–8.**
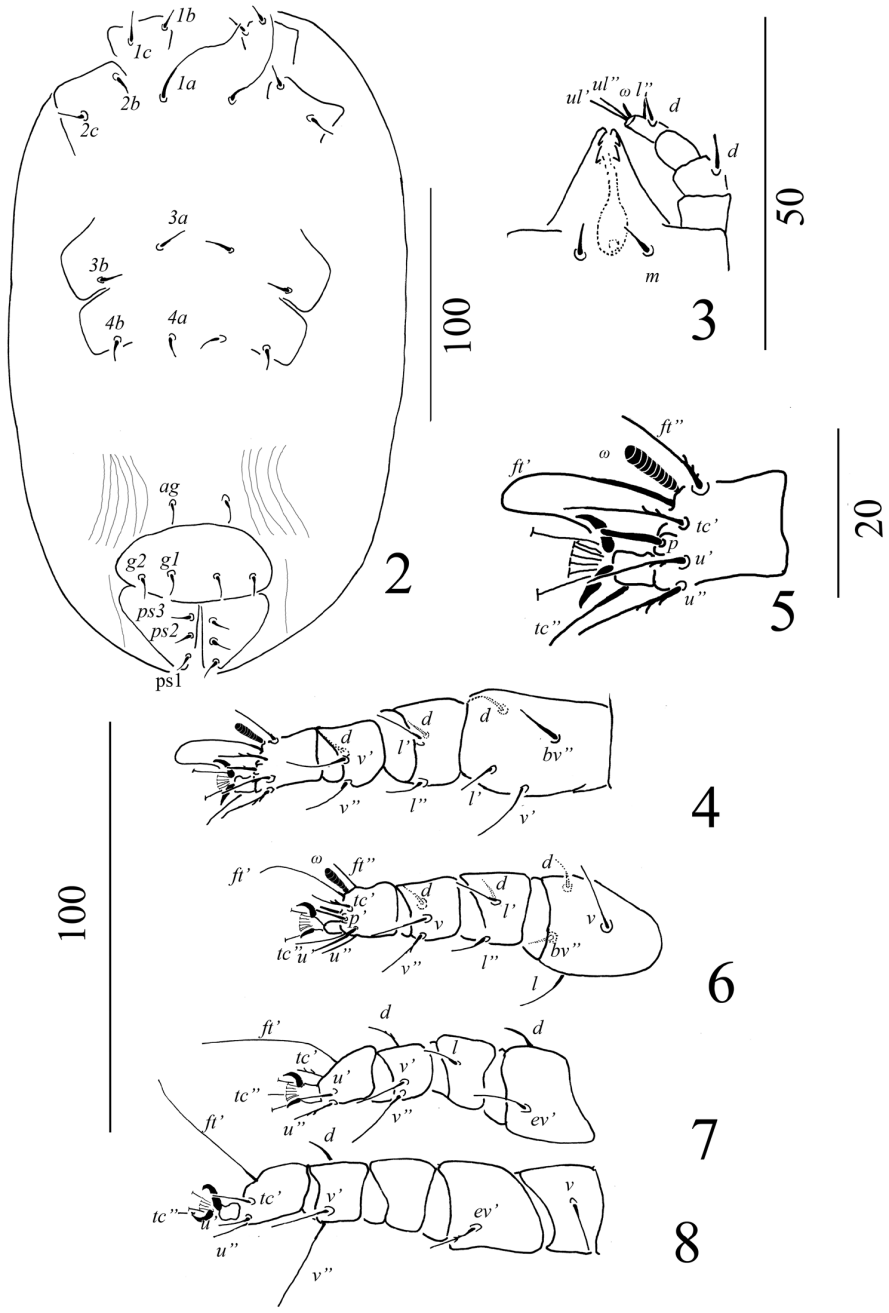
*Aegyptobiabozaii* sp. n., holotype, female **2** Ventral view of idiosoma **3** Ventral view of gnathosoma **4** Ventral view of leg I **5** Ventrolateral view of tarsus I **6** Ventral view of leg II **7** Ventral view of leg III **8** Ventral view of leg IV.

Venter (Figure 2): Surface of ventral idiosoma smooth, except lateral to *ag*, genital and anal plates where longitudinal striations visible. Genital and anal plates smooth. Length of ventral setae, *1a* 53–57, *3a* 11–12, *4a* 8–9, *1b* 7–8, *2b* 8–9, *3b* 8–9, *4b* 9–10, *1c* 11–12, *2c* 10–13, *ag* 8–9, *g1* 9–10, *g2* 7–8. Pseudanal setae, all 6–8. All ventral setae simple and smooth.

Gnathosoma (Figure 3): Rostrum extending to middle of tibia I; palp setal counts: tarsus with one solenidion and two eupathidia, tibia with two setae, genu without seta and femur with one simple dorsal seta. All setae smooth. Subcapitulum with setae *m* (4–5).

Legs (Figures 4–8): Setal formula for leg I-IV (coxae to tarsi): 3-1-4-3-3-9, 2-1-4-3-3-9, 2-2-2-1-3-5, 2-1-1-0-3-5. A supplementary lateral (*l*’) seta present on femora I. Solenidia on tarsi I and II 8–10 long, broad, leaf-like. Tarsal claws uncinate and empodium pad-like.

#### Deutonymph

**Figure 9.**
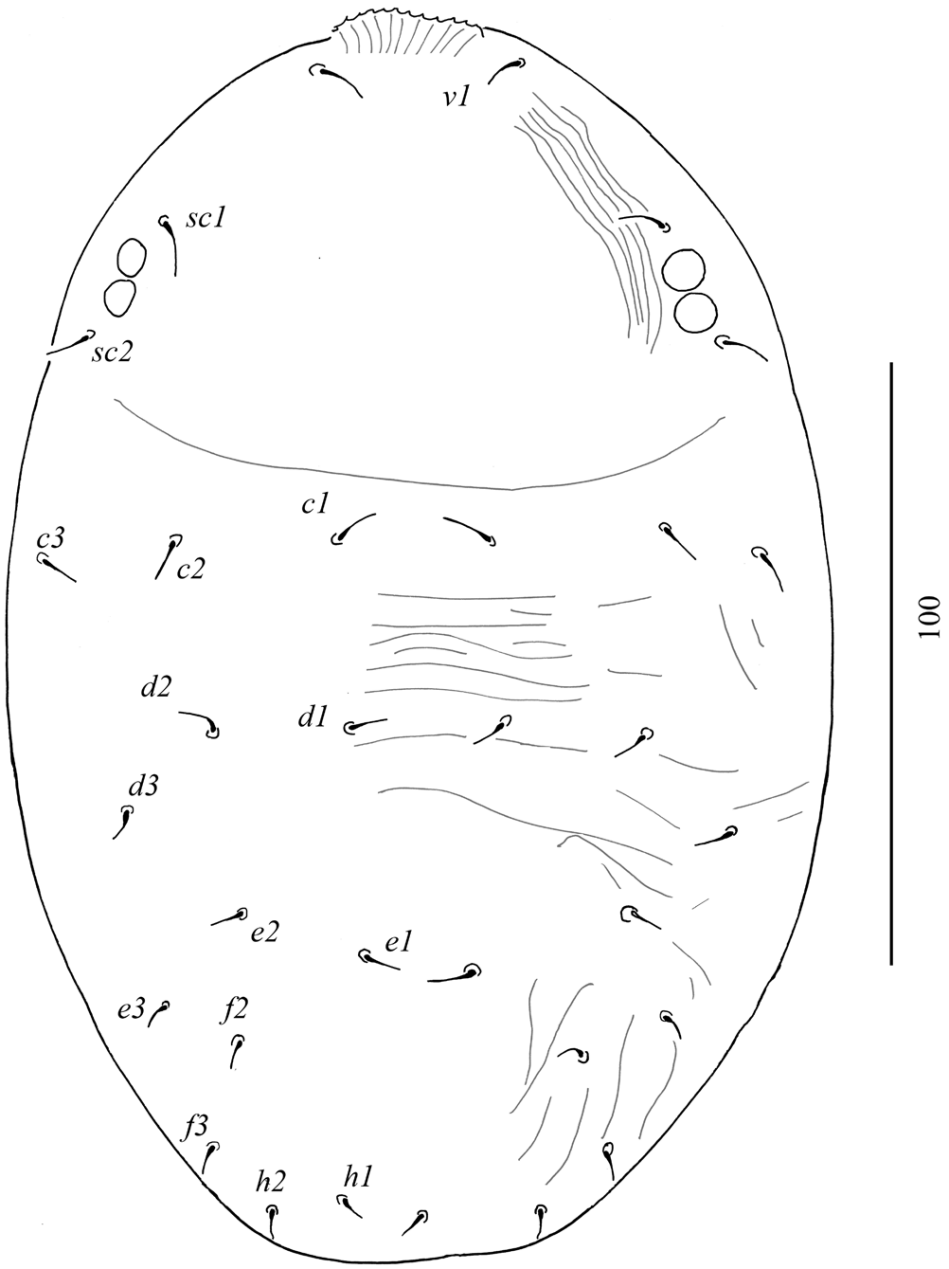
Dorsal view of *Aegyptobiabozaii* sp. n., paratype, deutonymph.

**Figures 10–15.**
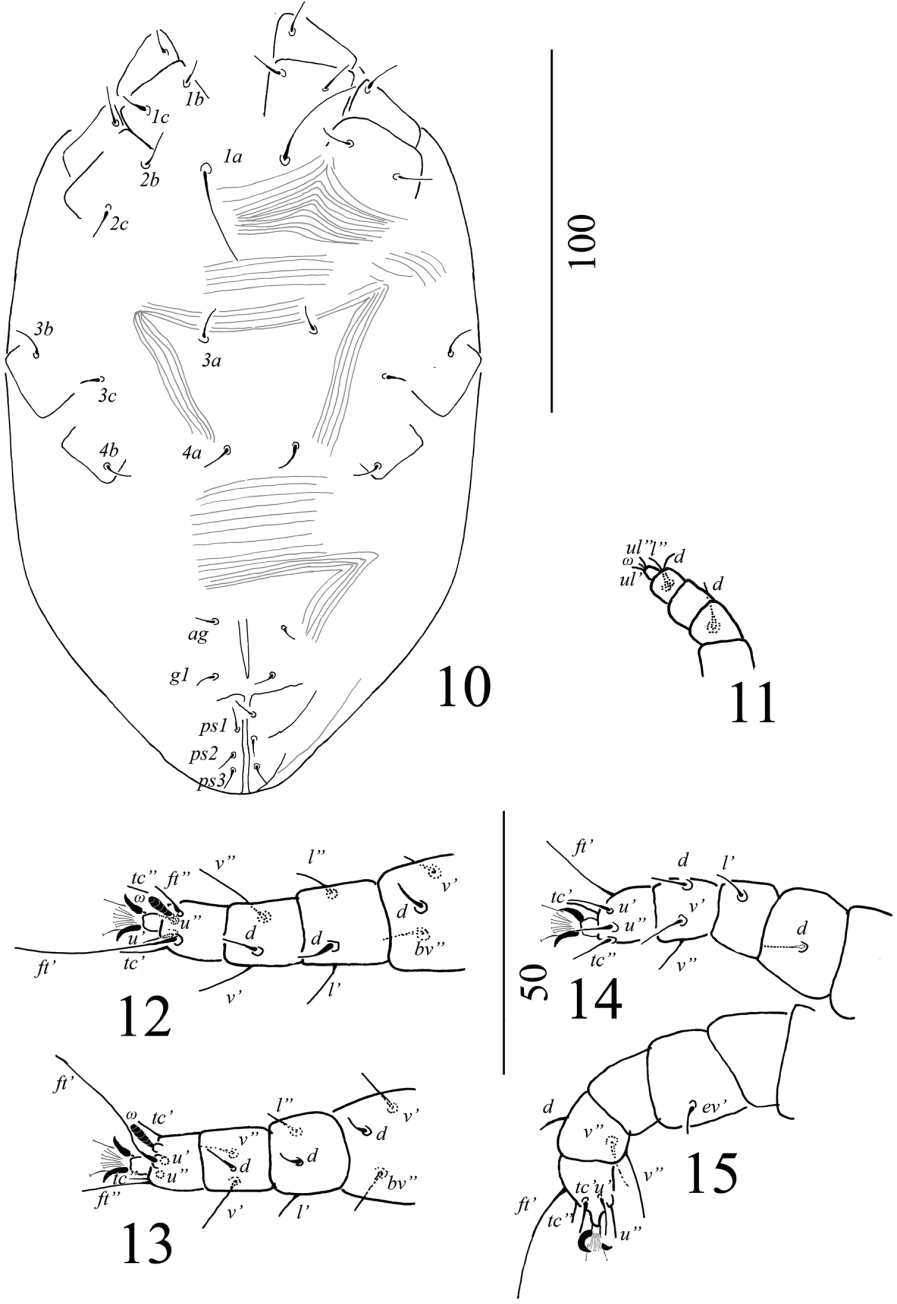
*Aegyptobiabozaii* sp. n., paratype, deutonymph **10** Ventral view of idiosoma **11** Ventral view of palp **12** Ventral view of leg I **13** Ventral view of leg II **14** Ventral view of leg III **15** Ventral view of leg IV.

(n = 3; Figures 9–15). Idiosoma oval in shape, body measured from *v2* to *h1* 190–200; width 130–140 near setae *sc2*.

Dorsum (Figure 9) covered with a few striae, all setae short, simple and needle-like. Length of all setae 5–7.

Venter covered with very few striae with one pair of setae *1a*, *1b*, *2b*, *3a*, *3b*, *3c*, *4a* and *4b*, one pair of aggenital, one pair of genital and three pairs of anal setae, all simple and smooth *1a* 15–16, other setae on venter 5–7 (Figure 10). Palp setal counts: tarsus with one solenidion and two eupathidia, tibia with two setae, genu without seta and femur with one simple dorsal seta (Figure 11). Legs as Figures 12–15 and Table 1.

#### Protonymph

**Figure 16.**
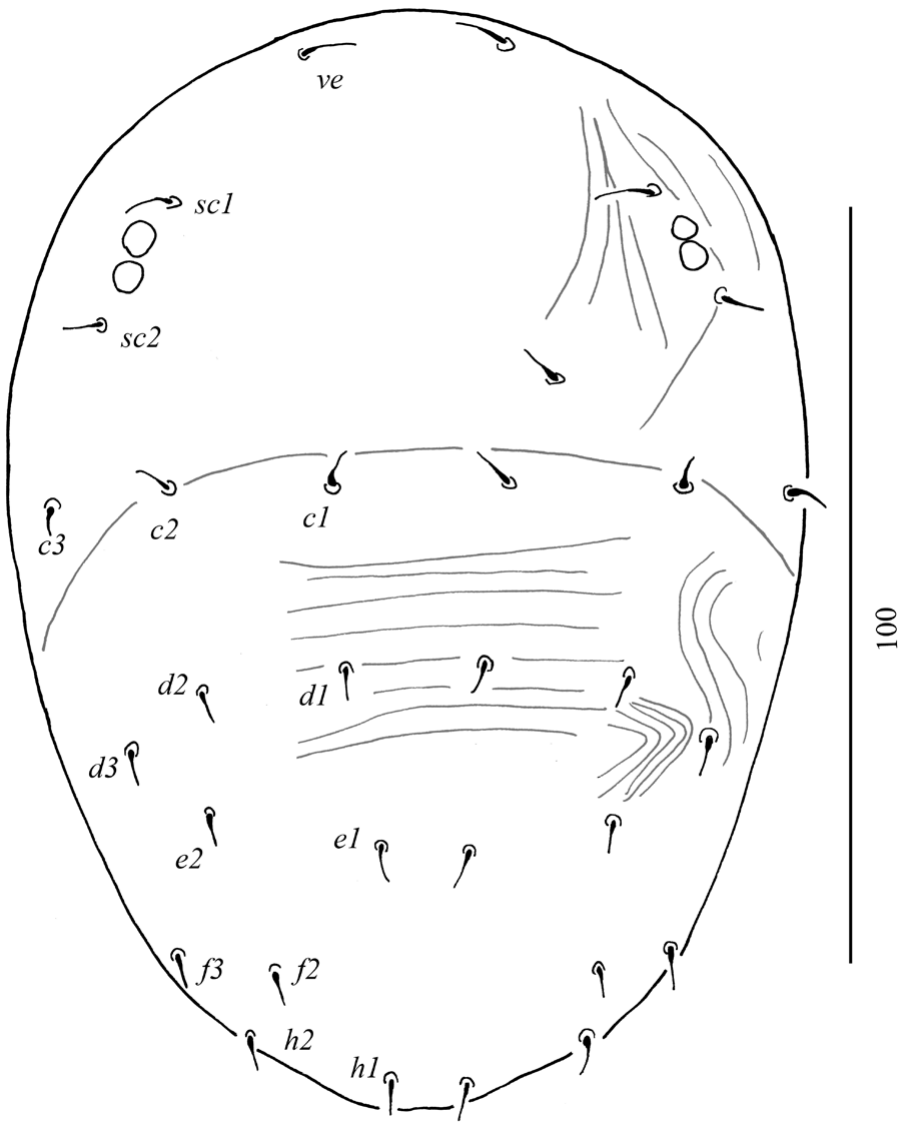
Dorsal view of *Aegyptobiabozaii* sp. n. paratype, protonymph.

**Figures 17–21.**
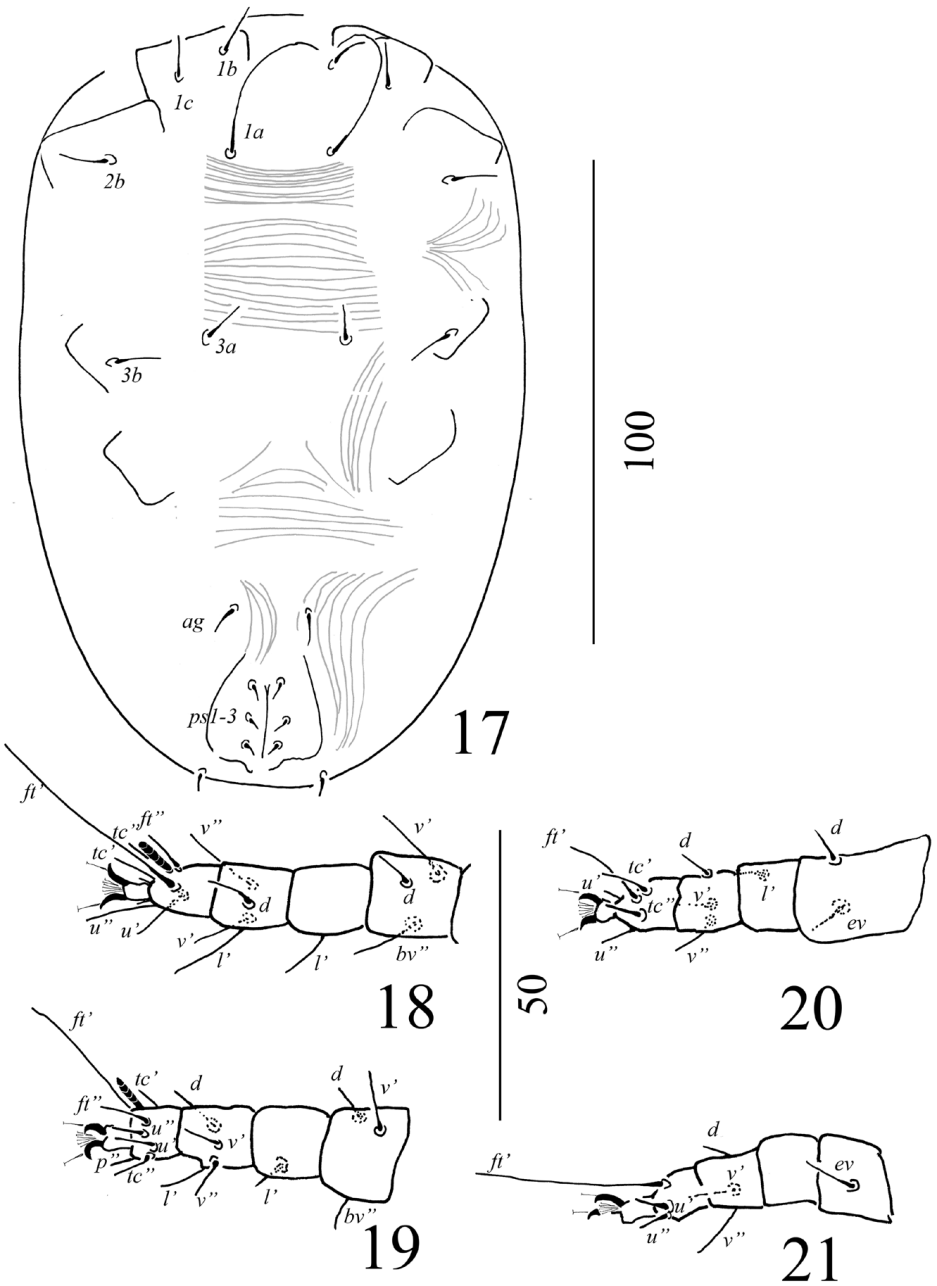
*Aegyptobiabozaii* sp. n., paratype, protonymph **17** Ventral view of idiosoma **18** Ventral view of leg I **19** Ventral view of leg II **20** Ventral view of leg III **21** Ventral view of leg IV.

(n = 3; Figures 16–21). Idiosoma oval in shape, body measured from *v2* to *h1* 149–155; width 94–100 near setae *sc2*.

Dorsum (Figure 16). Surface without striae, all setae short, simple and needle-like. All setae 4–6 in length.

Venter covered with very few striae with one pair of setae *1a*, *1b*, *2b*, *3a* and *3b*, one pair of aggenital and three pairs of anal setae, all simple and smooth. *1a* 13–15, other setae on venter 5–6 (Figure 17). Legs as Figures 18–21 and Table 1.

#### Larva

**Figure 22.**
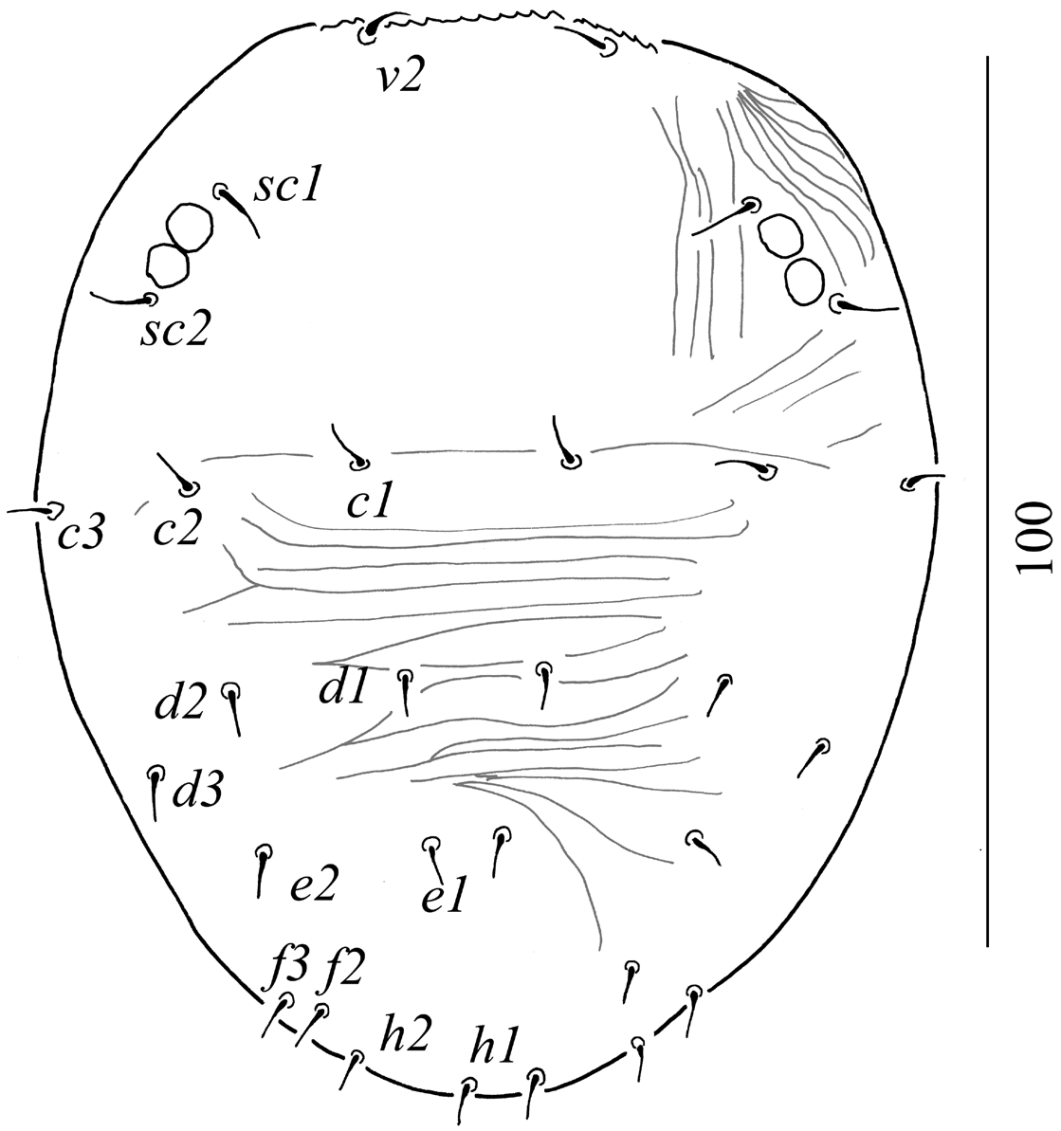
Dorsal view of *Aegyptobiabozaii* sp. n., paratype, larva.

**Figures 23–26.**
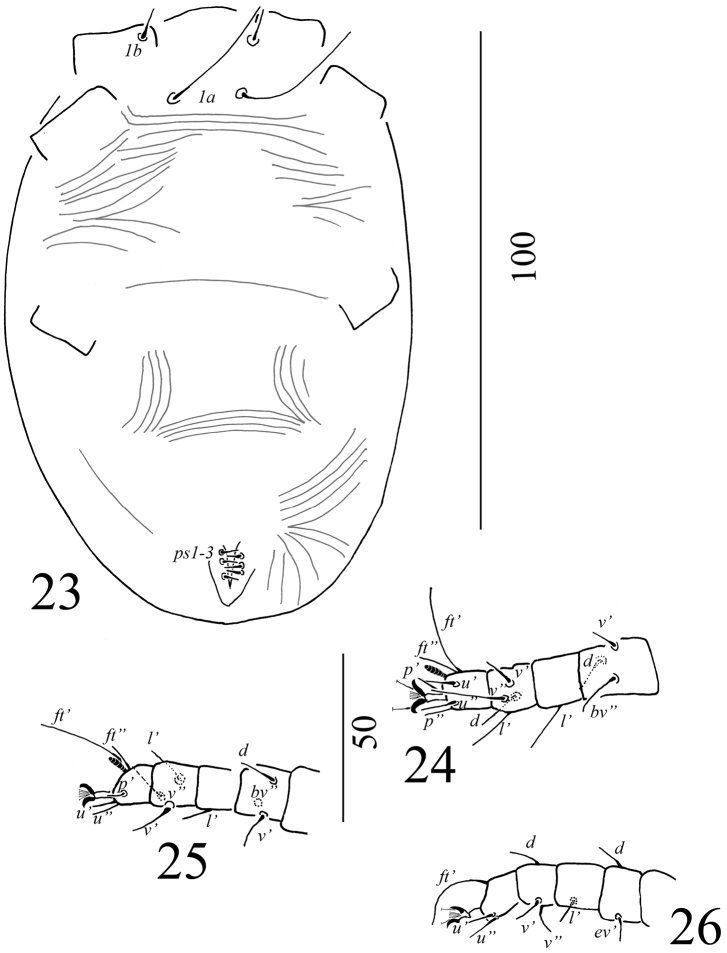
*Aegyptobiabozaii* sp. n., paratype, larva **23** Ventral view of idiosoma **24** Ventral view of leg I **25** Ventral view of leg II **26** Ventral view of leg III.

(n = 1; Figures 22–26). Idiosoma oval in shape, body measured from *v2* to *h1* 109; width 82 near setae *sc2*.

Dorsum (Figure 22). Covered with a few striae, all setae short, simple and needle-like. Length of setae: *v2* 30–31; *sc1* 30–37; *sc2* 28–36; *c1* 29–38; *c2* 26–39; *c3* 31–37; *d1* 5; d3 37–43; *e1* 3; *e3* 40–54; *f2* 3–4; *f3* 64–66; *h1* 2–3; *h2* 2–5.

Venter covered with few striae with one pair of setae *1a* and three pairs of anal setae, all simple and smooth (Figure 23). Legs as Figures 24–26 and Table 1.

**Table 1.** Development of leg setae (after Seeman and Beard 2011).

	**Cx I**			**Cx II**		**Cx III**		**Cx IV**		**Tr I**	**Tr II**	**Tr III**		**Tr IV**	Fe I*			**Fe II**			**Fe III**	
	1a	1b	1c	2b	2c	3a	3b	4a	4b	v’	v’	l’	v’	v’	d	v’	bv”	d	v’	bv”	d	ev’
Larva	+	+				+									+	+	+	+	+	+	+	+
PN	+	+	+	+		+	+					+			+	+	+	+	+	+	+	+
DN	+	+	+	+	+	+	+	+	+	+	+	+	+		+	+	+	+	+	+	+	+
Adult	+	+	+	+	+	+	+	+	+	+	+	+	+	+	+	+	+	+	+	+	+	+
	**Fe IV**	**Ge I**			**Ge II**			**Ge III**	Ti I**				**Ti II**				**Ti III**			**Ti IV**		
	ev’	l’	d	l”	l’	d	l”	l’	d	l’	v’	v”	d	l’	v’	v”	d	v’	v”	d	v’	v”
Larva		+			+				+	+	+	+					+	+	+	+	+	+
PN	+	+			+			+	+	+	+	+	+	+	+	+	+	+	+	+	+	+
DN	+	+	+	+	+	+	+	+	+	?	+	+	+	?	+	+	+	+	+	+	+	+
Adult	+	+	+	+	+	+	+	+	+	?	+	+	+	?	+	+	+	+	+	+	+	+
	**Ta I-II**									**Ta III**							**Ta IV**					
	u’	u”	p’	p”	tc’	tc”	ft’	ft”	ω	u’	u”	p’	p”	tc’	tc”	ft’	u’	u”	tc’	tc”	ft’	
Larva	+	+	+	+			+	+	+	+	+					+						
PN	+	+	+	+	+	+	+	+	+	+	+	+	+	+	+	+	+	+			+	
DN	+	+	+	+	+	+	+	+	+	+	+	+	+	+	+	+	+	+	+	+	+	
Adult	+	+	+	+	+	+	+	+	+	+	+	+	+	+	+	+	+	+	+	+	+	

* The new species has a supplementary lateral (*l*’) seta on femora I. ** The setae *l*’ on tibiae I and II in adult female and deutonymphs are not visible.

#### Etymology.

We dedicate the new species to Dr. József Bozai, former Hungarian tenuipalpid specialist.

#### Notes on the host.

The host plant, Hungarian statice (Limoniumgmeliniisubsp.hungaricum) (Plumbaginaceae), is an endemic subspecies occurring on salt meadows in Central-Hungary. The mites appeared to prefer leaves of the host plant that were lying close to the surface of the soil. The alkali steppe where the host plant was found is hot and dry in summer, typical habitat for tenuipalpid species, which prefer warm and dry conditions. Up until now, only one species has been reported from *Limonium* plants: *Capeduliamaritima* Gerson & Smith Meyer, 1980 was found on the roots of *Limoniummeyeri* in Israel (Ueckermann et al. 2018).

#### Remarks.

The new species has uncinate claws and therefore belongs to the *Aegyptobiatragardhi* species group (Khanjani et al. 2008). It is very similar to *A.iranensis*Khanjani et al., 2008 and *A.wainsteini* Bagdasarian, 1962 based on the claw-like empodium, the slender prodorsal setae, the deeply emarginated notch and the medially smooth prodorsum. The most important differences among three species are summarized in Table 2.

Only one species, *Aegyptobiawainsteini* Bagdasarian, 1962, was previously reported from Hungary from a *Biotaorientalis* tree (Cupressaceae) close to the town Kecskemét (Bozai 1969). Other new occurrences have not been given since this first report. The two *Aegyptobia* species reported from Hungary differ in the shape of the dorsal setae, which are short and smooth in *A.bozaii* sp. n. and longer and finely pilose on *Aegyptobiawainsteini*.

**Table 2.** Distinguishing characteristics among *Aegyptobiabozaii, A.iranensis* and *A.wainsteini*.

Character	* Aegyptobia bozaii *	* Aegyptobia iranensis *	* Aegyptobia wainsteini *
Surface between *c1* and *d1*	with large reticulations	with large reticulations	smooth
Distance between setae *v2*	three times longer than length of *v2*	two times longer than length of *v2*	same as length of *v2*
Setae *3a*	1/2 the distance *3a-3a*	two times the distance *3a-3a*	1/2 the distance *3a-3a*

### Tenuipalpus szarvasensis

Taxon classificationAnimaliaProstigmataTenuipalpidae

Bozai, 1970

Tenuipalpus szarvasensis Bozai, 1970: 367.
Tenuipalpus cheladzeae Gomelauri, 1960 as senior synonym of T.szarvasensis by Mitrofanov and Strunkova 1979: 51.
Tenuipalpus cheladzeae : Kontschán and Ripka 2017.

#### Material examined.

Holotype: female, HNHM Astig-242, Szarvas, 8 October 1968, from *Piceaexcelsa* Lk. No. 1250, Bozai, J. coll.

#### Diagnosis

(based on female). Anterior margin of prodorsal shield with forked projection; prodorsum smooth medially, with some striae laterally; anterolateral projections carrying setae *sc2* weakly formed. Opisthosoma smooth anteriorly, with posteromedial reticulation and posterolateral longitudinal striation. Propodosomal setae as follows: *v2* short and smooth, *sc1* broad and obovate, *sc2* long and phylliform. Opisthosomal setae: *c1, c3* and *d1* broad, long, oblanceolate, *d3* short and oblanceolate, *e1* short and smooth, *h1, f1, f2* and *e3* long, oblanceolate, *h2* very long and smooth. Rostrum extending to middle of tibia I. Genital flap smooth. Intercoxal area between *3a* and *4a* smooth, *1a* and *4a* very long, *1b, 2b, 2c, 3a, 3b, 4b, ag, g1, g2* short. Legs with large, broad and phylliform, smooth and pilose setae.

#### Description

**Figure 27.**
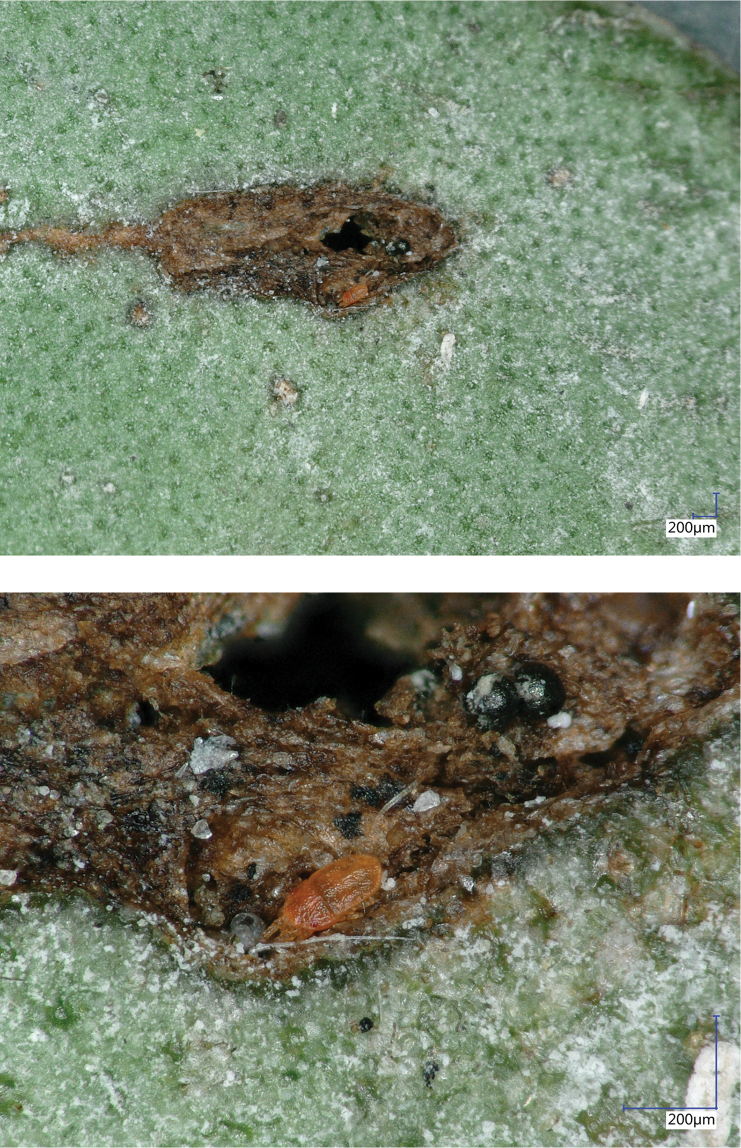
Photos of the female of *Aegyptobiabozaii* sp. n., paratype.

(female holotype). Colorization of idiosoma not observable in the holotype. Idiosoma (Figure 27) pentagonal in shape, body measured from *v2* to *h1* 305; from tip of rostrum 350; width between setae *sc2* 190.

Dorsum (Figure 29): Anterior margin of prodorsal shield with paired projections, depth of notch 16. Prodorsum smooth medially, with some striae laterally; anterolateral projections carrying setae *sc2* weakly formed. Opisthosoma smooth anteriorly, with posteromedial reticulation and posterolateral longitudinal striation. Propodosomal setae as follows: *v2* short and smooth, *sc1* broad and obovate, *sc2* long and phylliform. Opisthosomal setae: *c1, c3* and *d1* broad, long, oblanceolate, *d3* short and oblanceolate, *e1* short and smooth, *h1, f1, f2* and *e3* long, oblanceolate, *h2* very long and smooth. Rostrum extending to middle of tibia I. Opisthosomal pores between *d1* and *e1*. Length of dorsal setae: *v2* 9–10, *sc1* 30–32, *sc2* 65, *c1* 45–46, *c3* 32–34, *d1* 38–40, *d3* 12–14, *e1* 12, *e2* 45–47, *f2* and *f3* 45–46, *h1* 36–37, *h2* 105–110.

**Figure 28.**
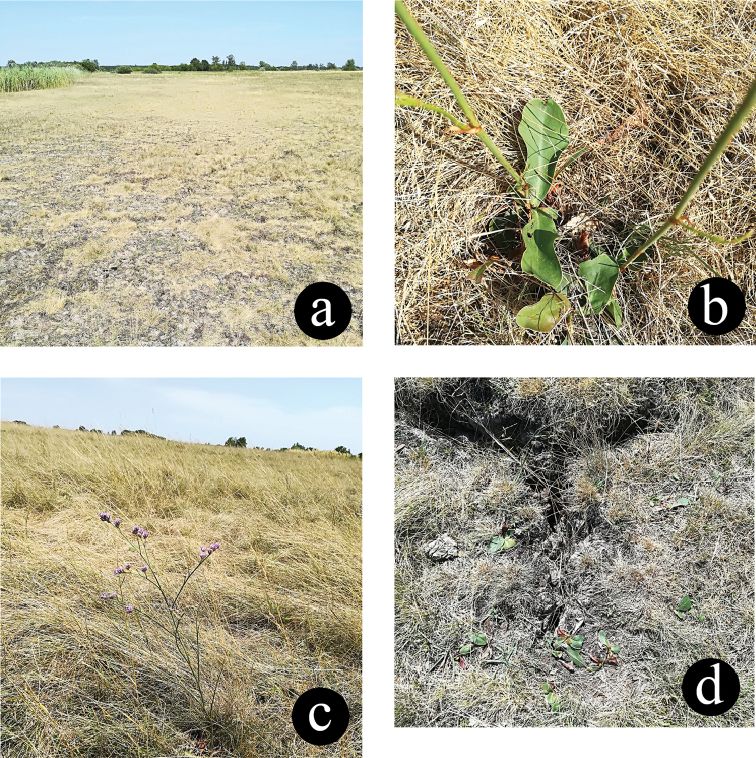
Habitat and details of the host plant. **a** Habitat **b** Host plant **c–d** Leaves of host plant.

**Figure 29.**
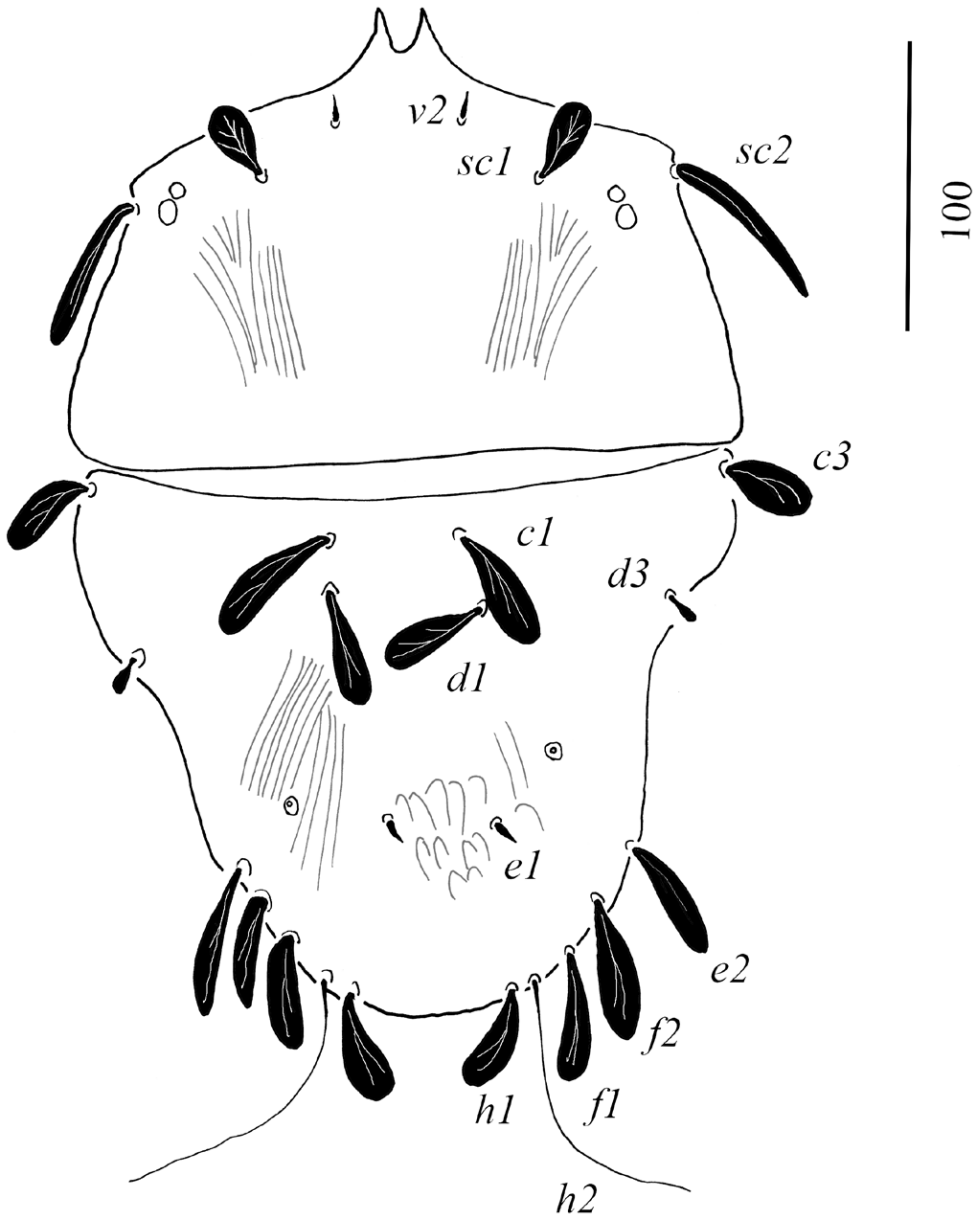
Dorsal view of *Tenuipalpusszarvasensis* Bozai, 1970, holotype, female.

**Figures 30–35.**
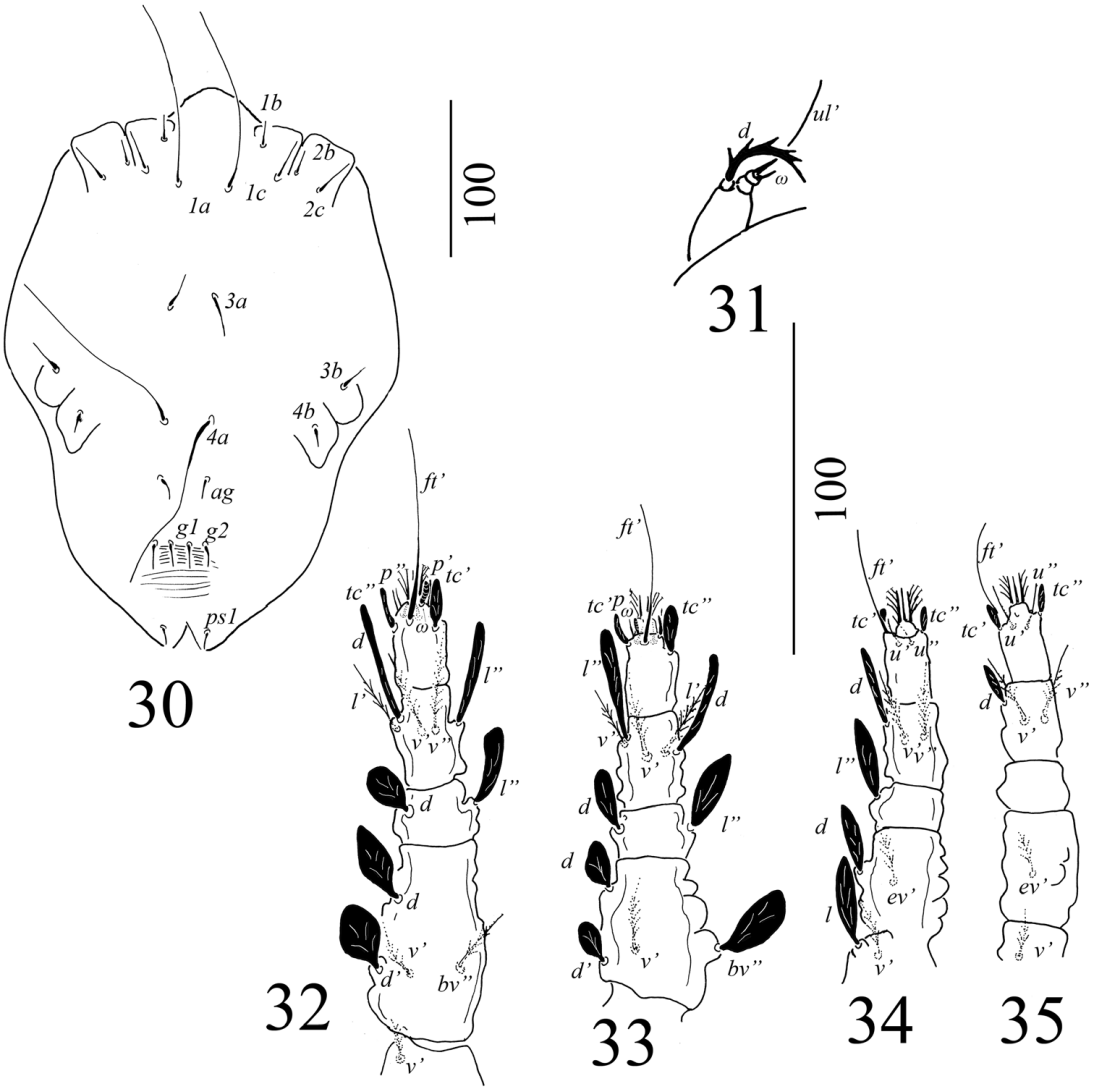
*Tenuipalpusszarvasensis* Bozai, 1970, holotype, female **30** Ventral view of idiosoma **31** Dorsal view of palp **32** Ventral view of leg I **33** Ventral view of leg II **34** Ventral view of leg III **35** Ventral view of leg IV.

Venter (Figure 30): Very few striations observable in the holotype, only a few longitudinal striations visible posterior to *g1*–*g2*. Genital and anal plates smooth. Length of ventral setae, *1a* 120–122, *3a* 26–27, *4a* 130–133, *1b* 16, *2b* 16–17, *3b* 18–19, *4b*13, *1c* and *2c* 24–25, *ag* 12, *g1*–*g2* 14–16. Pseudanal setae, all 12–13. All ventral setae simple and smooth.

Gnathosoma: Rostrum extending to middle of tibia I; palp setal counts as in Figure 31.

Legs: Setal formula for leg I–IV (coxae to tarsi): 3-1-4-2-5-9, 2-1-4-2-4-9, 2-2-2-1-3-5, 2-1-1-0-3-5. Shape of the setae on legs illustrated on Figures 32–35.

#### Notes.

Bozai’s (1970) noted in his detailed description of *Tenuipalpusszarvasensis*, that the species is very similar to *T.cheladzeae*, but he mentioned some easy to observe differences (like shape and length of setae *c1, d1*) between these two species. Despite these known differences, Mitrofanov and Strunkova (1979) synonymized the name under *Tenuipalpuscheladzeae*. Mitrofanov and Strunkova (1979) did not study the type specimens of *T.szarvasensis*, therefore their opinion was questionable.

**Figures 36–37.**
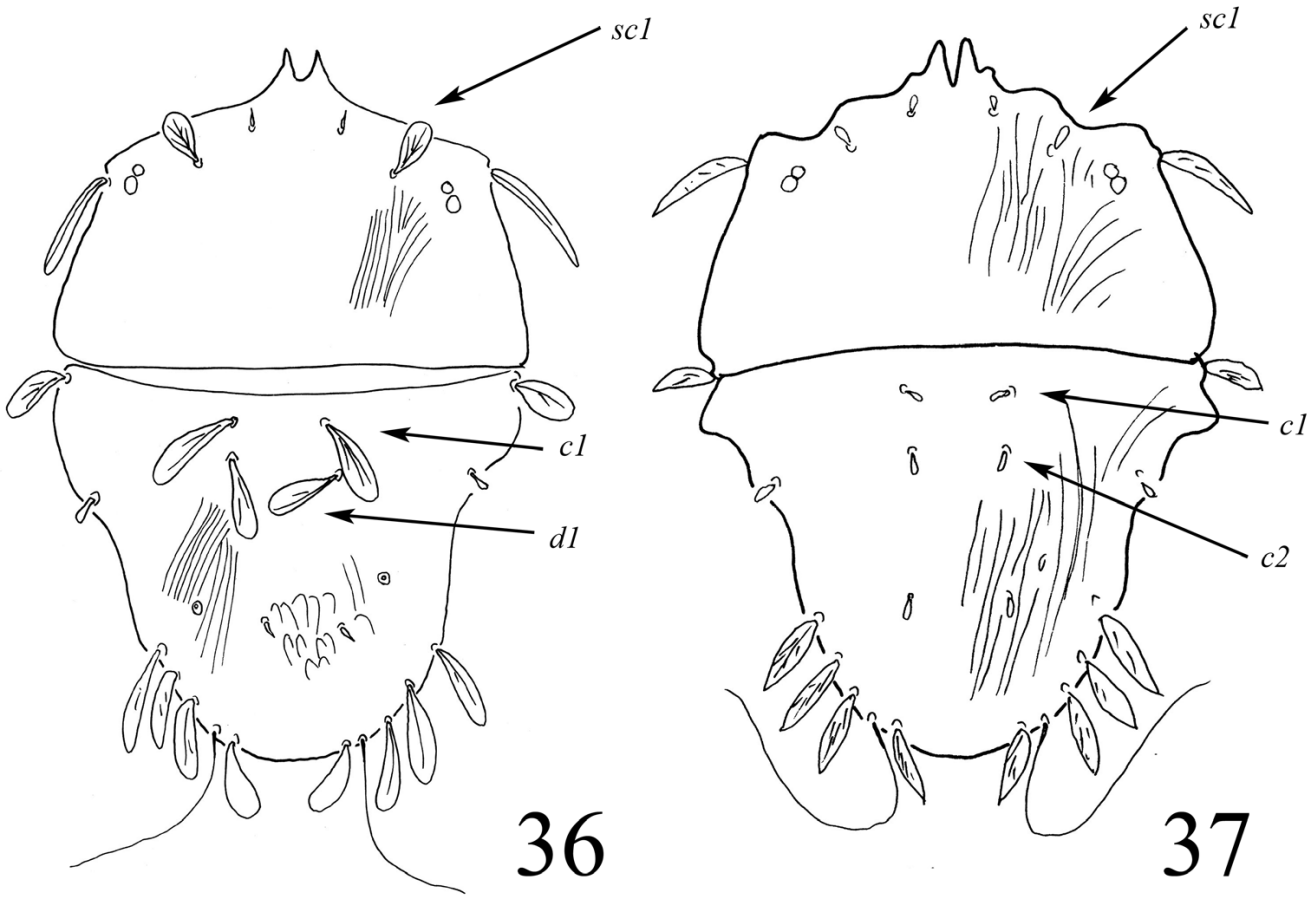
Illustrated differences between *T.szarvasensis* (**36**) and *T.cheladzeae* (**37**) Drawing of *T.cheladzeae* modified after Mitrofanov and Strunkova (1979).

This year, we studied the types of Bozai’s *T.szarvasensis* in order to confirm Bozai’s hypothesis that *T.szarvasensis* differs from Gomelauri’s *T.cheladzeae*. The differences are presented in Table 3 and are illustrated in Figures 36, 37.

*Tenuipalpus* is the largest genus of flat mites, but very few are known from Pinaceae. Apart from the above-mentioned two species, the only other species is *T.hondurensis* Evans, in Evans et al. (1993) (Mesa et al. 2009), which is considerably different from both the above species. *Tenuipalpuscupressoides* Smith, Meyer & Gerson, 1980 is very similar to *T.szarvasensis*, but the shape of *v2* and *e2* and the length of *sc1, c1* and *d1* are different. In addition, the host plant, *Cupressussempervirens* of *T.cupressoides* belongs to the family Cupressaceae and not to Pinaceae (Ueckermann et al. 2018).

**Table 3.** Distinguishing characters between *Tenuipalpuscheladzeae* and *T.szarvasensis*.

Character	* T. cheladzeae *	* T. szarvasensis *
Shape of *v2*	apically rounded	apically pointed
Shape of *sc1*	short (12–14) and bulbiform	long (30–32) and phylliform
Shape of *c1* and *d1*	short (*c1* 10–11, *d1* 9–11) and bulbiform	long (*c1* 45–46, *d1* 38–40) and phylliform

## Supplementary Material

XML Treatment for Aegyptobia bozaii

XML Treatment for Tenuipalpus szarvasensis
